# Development of the Early Axon Scaffold in the Rostral Brain of the Small Spotted Cat Shark (*Scyliorhinus canicula*) Embryo

**DOI:** 10.1155/2014/196594

**Published:** 2014-10-28

**Authors:** Michelle Ware, Colin P. Waring, Frank R. Schubert

**Affiliations:** ^1^Institute of Biomedical and Biomolecular Science, University of Portsmouth, PO1 2DY Portsmouth, UK; ^2^Institut de Génétique et Développement, CNRS, UMR6290, Faculté de Médecine, Université de Rennes 1, 35000 Rennes, France; ^3^Institute of Marine Sciences, School of Biological Sciences, University of Portsmouth, P04 9LY Portsmouth, UK

## Abstract

The cat shark is increasingly used as a model for Chondrichthyes, an evolutionarily important sister group of the bony vertebrates that include teleosts and tetrapods. In the bony vertebrates, the first axon tracts form a highly conserved early axon scaffold. The corresponding structure has not been well characterised in cat shark and will prove a useful model for comparative studies. Using pan-neural markers, the early axon scaffold of the cat shark, *Scyliorhinus canicula*, was analysed. Like in other vertebrates, the medial longitudinal fascicle was the first axon tract to form from a small cluster of neurones in the ventral brain. Subsequently, additional neuronal clusters and axon tracts emerged which formed an array of longitudinal, transversal, and commissural axons tracts in the *Scyliorhinus canicula* embryonic brain. The first structures to appear after the medial longitudinal fascicle were the tract of the postoptic commissure, the dorsoventral diencephalic tract, and the descending tract of the mesencephalic nucleus of the trigeminal nerve. These results confirm that the early axon scaffold in the embryonic brain is highly conserved through vertebrate evolution.

## 1. Introduction

The initial nerve connections that develop during early development of the rostral brain form a structure that has been termed the early axon scaffold [1]. This structure has remained highly conserved through the vertebrate taxa. These tracts act as pioneers for the later, follower axons and allow more complex connections to be made. The early axon scaffold has been well described in non-jawed vertebrates such as lamprey [2] and jawed, bony vertebrates such as zebrafish,* Xenopus*, chick, and mouse [1, 3–5]. Briefly, the early axon scaffold forms from small clusters of neurones in distinct regions of the brain which project axons forming longitudinal, transversal, and commissural tracts. In all vertebrates studied, apart from mouse, the medial longitudinal fascicle (MLF) forms first, from a cluster of neurones in the basal diencephalon. In mouse, the descending tract of the mesencephalic nucleus of the trigeminal nerve (DTmesV) neurones appears first, closely followed by the appearance of MLF neurones [4, 6]. In anamniotes, the prosencephalon contains an array of tracts and commissures, such as the anterior commissure (AC), supraoptic commissure (SOT), postoptic commissure (POC), and tract of the postoptic commissure (TPOC).

Analysis of cartilaginous fish is particularly interesting as this is the sister group to bony vertebrates. Similarities between the two branches of gnathostomes provide an insight into the last common ancestor of jawed vertebrates. For this reason the group is often utilised in origin and evolution studies by comparative embryologists. In this study, the small spotted cat shark (or lesser spotted dog shark), an emerging model for studying and comparing brain development, has been used. While the adult brain connections have been well described in cartilaginous fish, a description of the initial axon tracts is lacking [7]. Neuronal development of specific neurone types has been studied in this cat shark but at later stages after the early axon scaffold has formed [8–12]. The early axon scaffold has been mentioned in the cat shark species,* Scyliorhinus torazame,* but this study only showed the formation at an older stage (Stage V) once the early axon scaffold appeared to be fully established [13].

The aim of this study was to characterise the early axon scaffold through a time series in the cat shark species,* Scyliorhinus canicula*, using immunostaining.

## 2. Materials and Methods

### 2.1. *S. canicula *Embryos

Sexually mature cat sharks (*S*.* canicula*) were caught locally in the summer off the coast of Portsmouth, Hampshire, UK, and kept in a flow-through 500 L seawater tank under ambient conditions at the Institute of Marine Sciences, University of Portsmouth. Females regularly laid eggs wrapped around the outflow pipes of the tank.* S. canicula* eggs were incubated in flow-through seawater at approximately 17°C until they reached the required embryonic stage [14]. To harvest the embryo, a window was cut into the top egg to allow access. The embryos were washed in phosphate buffered saline (PBS) and fixed with 4% paraformaldehyde/PBS or MEMFA (0.1 M MOPS/2 mM EGTA/1 mM MgSO_4_/3.7% formaldehyde; 30–40 minutes, washed and stored in methanol).

### 2.2. Immunohistochemistry

*S. canicula* embryos were prepared for immunofluorescence by opening the rhombencephalon and the telencephalic vesicles. The axon tracts were visualised using pan-neural antibodies, Tuj1 mouse (Abcam ab7751; 1 : 1000) or rabbit (Abcam ab18207; 1 : 1000), and the neuronal populations specifically with anti-HuC/D (Molecular Probes A21271; 1 : 500). Primary antibodies were visualised with Alexa fluorochrome-conjugated IgG anti-mouse (Invitrogen A11001; 1 : 500) or anti-rabbit antibody (Invitrogen A11008; 1 : 500). The protocol for immunohistochemistry has been described previously [15].

### 2.3. DiO Labeling

Cyanine dye, DiO (3,3′-dioctadecyloxacarbocyanine perchlorate, Molecular Probes, D275), was used to trace the MLF axons from the rhombencephalon.* S. canicula* embryonic brains were injected with dye as previously described [5].

### 2.4. Microscopy and Image Processing

A Zeiss Stereo Lumar V12 fluorescent stereomicroscope was used to obtain low magnification images of the embryos. For more detailed images, Zeiss LSM 510 and LSM 710 confocal microscopes were used. Images were processed using Axiovision Rel. 4.6, ImageJ and Photoshop CS software.

## 3. Results

*S. canicula* embryos have been studied between stages 18 and 25 [14]. In terms of early axon development these stages were equivalent to zebrafish stages 16 hours post fertilisation (hpf) to 24 hpf,* Xenopus* stages 22 to 32, chick stages HH11 to HH18, and mouse stages E8.5 to E10.5 (data not published).

### 3.1. Detailed Description of the First Neurones to Differentiate in the Embryonic* S. canicula* Brain

**Figure 1:**
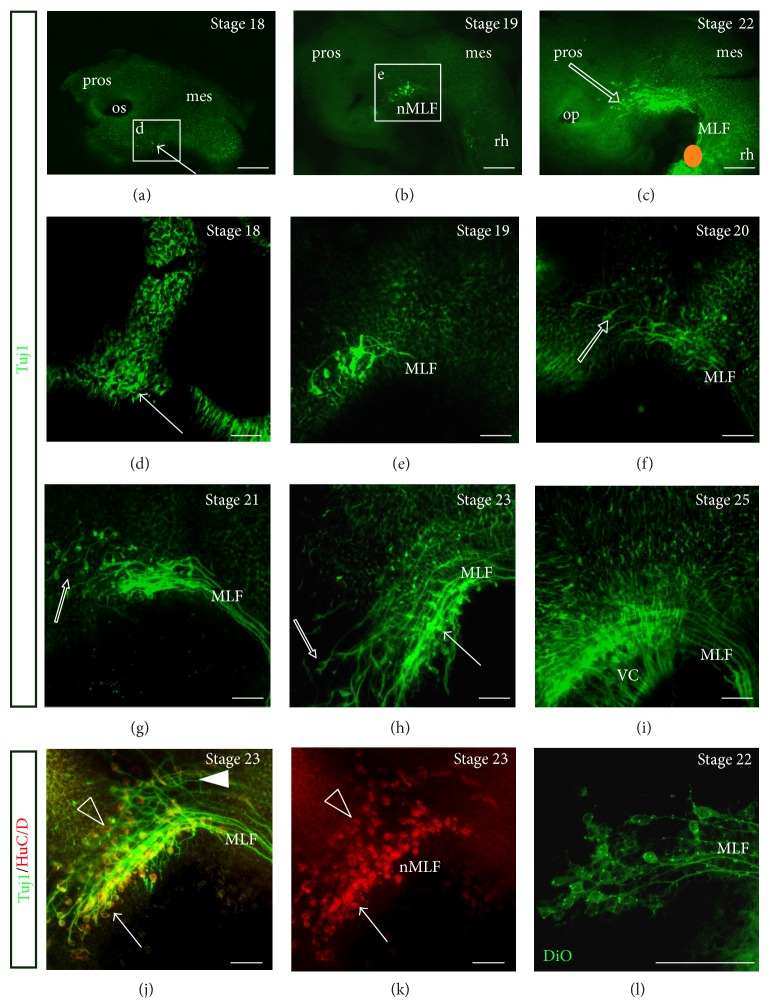
Detailed formation of the MLF in the embryonic* S. canicula* brain. Lateral view of whole mount embryonic brain. Scale bars, 100 *μ*m. (a)–(c) Overview of embryos. (a) Stage 18. The first neurones appeared at the ventral DMB (arrow). These neurones formed the nMLF. Box indicates high magnification in D. (b) Stage 19. The number of MLF neurones has increased. Box indicates high magnification in E. (c) Stage 22. The MLF was still the prominent tract, with scattered neurones located rostrally projecting axons into the MLF (unfilled arrow). Orange spot indicated where DiO was injected. (d) Stage 18. The first MLF neurones appeared (arrow). (e) Stage 19. The MLF neurones were projecting their first axons caudally. (f) Stage 20. The MLF axons have projected further caudally and the first rostrally located; scattered MLF neurones appeared (unfilled arrow). (g) Stage 21. The MLF axons have formed a tight bundle. The scattered MLF neurones were projecting into the MLF tract (unfilled arrow). (h) Stage 23. The MLF has increased in number of axons and neurones. Scattered MLF neurones projected into the MLF axon tract (unfilled arrow). A tight population of MLF neurones was present ventrally (arrow). (i) Stage 25. The MLF was well established and the VC was also present. (j) Stage 23, double labelling with Tuj1 and HuC/D. Caudal to the scattered MLF neurones, the MLF nucleus appeared to be arranged into a further two populations of MLF neurones: one located dorsally (unfilled arrowhead) and the other as a tight cluster of centrally located neurones along the ventral midline (arrow). There were also axons projecting ventrally to the MLF (arrowhead) that appeared to be a separate axon tract. (k) HuC/D labelling only of (j). (l) Stage 22. The MLF labelled with DiO. DiO was injected into the rhombencephalon close to the ventral midline to label MLF axons. Mes: mesencephalon; MLF: medial longitudinal fascicle; nMLF: nucleus of the medial longitudinal fascicle; op: olfactory pit; os: optic stalk; pros: prosencephalon; rh: rhombencephalon; tel: telencephalon; VC: ventral commissure.

**Figure 2:**
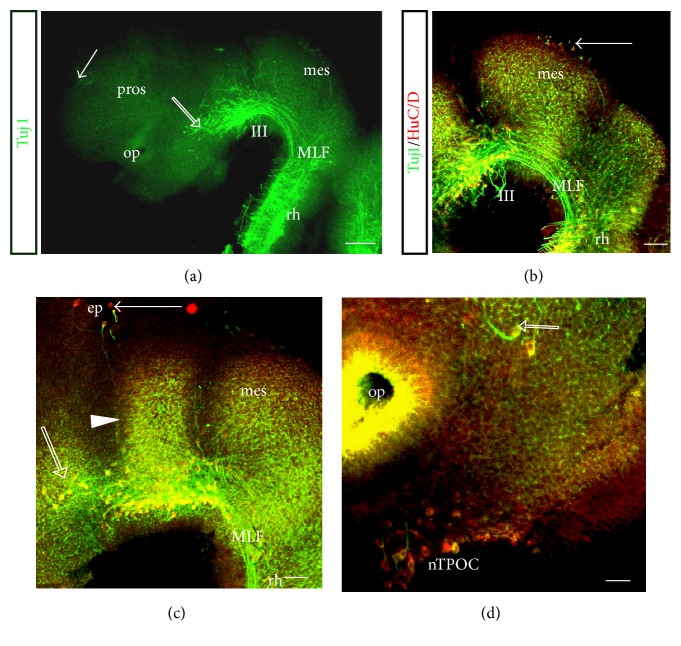
Development of axon tracts in the embryonic* S. canicula* brain. Lateral view of whole mount embryonic brain. Scale bars, 100 *μ*m. (a) Stage 23, overview. The MLF neurones have projected well into the rhombencephalon. The scattered MLF neurone population has increased in size (unfilled arrow). The first neurones were present in the dorsal prosencephalon (arrow). (b)–(d) Stage 23, double labelled with Tuj1 (green) and HuC/D (red). (b) Neurones differentiated along the dorsal midline of the mesencephalon to give rise to the DTmesV (arrow). The MLF axon tract was well formed. (c) Three-four neurones appeared dorsally at the epiphysis (arrow). One neurone had projected its axon ventrally, almost reaching the MLF (arrowhead). This axon was a pioneer of the DVDT. Scattered MLF neurones were located rostrally to the initial MLF neurones (unfilled arrow). (d) Basal hypothalamus, where the TPOC neurones (nTPOC) differentiated. Scattered MLF neurones (unfilled arrow). ep: epiphysis; mes: mesencephalon; MLF: medial longitudinal fascicle; nTPOC: nucleus of the tract of the postoptic commissure; op: olfactory pit; pros: prosencephalon; III: oculomotor nerve.

Using an antibody against *β*III tubulin (Tuj1), the first neurones were detected within the* S. canicula* embryonic brain at stage 18 (Figure 1(a), d, arrow). These neurones will form the nucleus of the MLF (nMLF) and occupied a location similar to the MLF neurones in the chick brain [5], presumably rostral to the diencephalic-mesencephalic boundary (DMB). The MLF neurones started projecting axons caudally at stage 19 (Figure 1(b), e). The MLF continued to project axons caudally at stage 20 (Figure 1(f)). Also evident at stage 20 (Figure 1(f), unfilled arrow) and stage 21 (Figure 1(g), unfilled arrow) were a scattered population of neurones located more rostrally compared with the initial MLF neurones, but still projecting axons into the MLF tract. By stage 21 the MLF had formed a tight bundle that projected along the floor plate into the rhombencephalon (Figure 1(g) and data not shown). To confirm that the MLF had reached the rhombencephalon, the MLF axon tract was labelled with DiO from the rhombencephalon at stage 22 (Figure 1(c), orange spot). The dye diffused back to neurones located in the diencephalon that belonged to the nMLF (Figure 1(l)). This confirmed that the axons of the MLF had reached the rhombencephalon by stage 22. At stage 23, the MLF was well established and appeared to be formed from three populations of neurones (Figures 1(h), 1(j), and 1(k)). Double labelling with Tuj1 and HuC/D revealed one population that was located along the floor plate (Figures 1(h), 1(j), and 1(k), arrow), another population that was located more dorsally (Figures 1(j) and 1(k), unfilled arrowhead), and the rostrally located scattered neurones (Figure 1(h), unfilled arrow). The population of neurones along the floor plate were tightly clustered and appeared to be contributing most of the axons to the MLF, while the dorsal population was more scattered, like the more rostral population. There were axons projecting dorsally to the MLF axon tract and appeared to be a separate tract (Figure 2(j), arrowhead). These axons most likely projected from neurones that were part of the rostral MLF scattered population. By stage 25, the MLF was well established and the ventral commissure (VC) had formed across the ventral midline (Figure 1(i)).

### 3.2. Formation of the DTmesV, DVDT, and TPOC in the Embryonic* S. canicula* Brain

**Figure 3:**
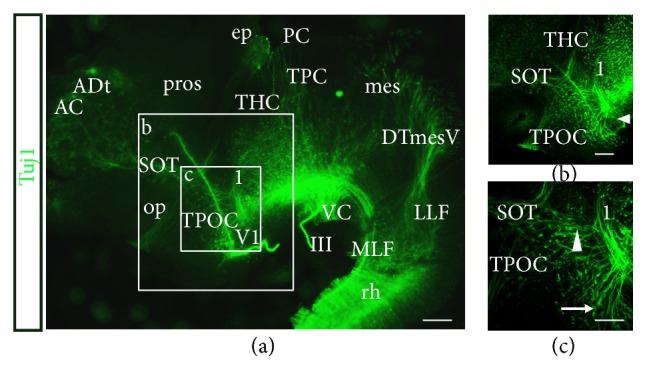
Description of the early axon scaffold at stage 25 in the embryonic* S. canicula* brain. Lateral view of whole mount embryonic brain. Scale bars, 100 *μ*m. (a) Overview of the early axon scaffold at stage 25. All axon tracts were well established. Boxes indicate magnified images in (b) and (c). (b) Prosencephalon. The TPOC, SOT, and THC were well established. An unidentified tract (1) was located caudally in the diencephalon. The neurones were possibly located at the ventral midline (small arrowhead). (c) Unidentified tract 1 appeared to be projecting dorsally before turning to project slightly caudally. Possible MTT neurones were projecting axons ventrally (filled arrow). The TPOC axons have turned at a right angle and project ventrally (arrowhead). AC: anterior commissure; ADt: anterior dorsal telencephalic neurones; DTmesV: descending tract of the mesencephalic nucleus of the trigeminal nerve; ep: epiphysis; LLF: lateral longitudinal fascicle; mes: mesencephalon; MLF: medial longitudinal fascicle; op: olfactory pit; PC: posterior commissure; pros: prosencephalon; rh: rhombencephalon; SOT: supraoptic tract; tel: telencephalon; THC: tract of the habenular commissure; TPC: tract of the posterior commissure; TPOC: tract of the postoptic commissure; V1: ophthalmic profundus nerve; VC: ventral commissure; III: oculomotor nerve.

Interestingly, until stage 23, the MLF was still the only prominent tract in the* S. canicula* brain (Figures 1(a)–1(c)). Although it was not clear from the overview image at stage 23 (Figure 2(a)), higher magnification with double labelling of Tuj1 and HuC/D antibodies revealed that there were other axon tracts beginning to form (Figures 2(b)–2(d)). Neurones appeared along the dorsal midline of the alar plate in the mesencephalon that formed the nucleus of the DTmesV (nmesV) giving rise to the DTmesV (Figure 2(b), arrow). There were 3-4 neurones located dorsally at the epiphysis (Figure 2(c), arrow). These neurones started projecting axons ventrally pioneering the dorsoventral diencephalic tract (DVDT). One of these neurones had projected an axon ventrally almost reaching the MLF (Figure 2(c), arrowhead). The tract of the postoptic commissure (TPOC) formed from neurones located in the rostral hypothalamus, the nucleus of the TPOC (nTPOC) (Figure 3(d)). There were also neurones located in the dorsal telencephalon that will eventually give rise to the anterior dorsal telencephalic (ADt) population (Figure 2(a), arrow).

### 3.3. Detailed Description of the Established Early Axon Scaffold in the Embryonic* S. canicula* Brain

Between stages 23 and 25, the early axon scaffold developed rapidly. The number of axon tracts present increased and the early axon scaffold was very well established and likely contained later, follower axons that were using the scaffold for guidance (Figure 3(a)). From its origin close to the DMB, the MLF extended caudally along the floor plate into the rhombencephalon (Figure 3(a)). The DTmesV neurones projected axons from the dorsal midline of the mesencephalon first ventrally; then the axons turned caudally to pioneer the lateral longitudinal fascicle (LLF) that projected into the rhombencephalon (Figure 3(a)). In the rostral hypothalamus, the TPOC neurones first projected axons dorsally, while remaining ventral to the olfactory pits, before turning almost at a right angle to project caudally once the axons reached the SOT (Figure 3(c), arrowhead). The dorsal telencephalon contained a large, mixed population of neurones, the ADt (Figure 3(a)). These neurones appeared to be homologous to the dorsorostral cluster (drc) in zebrafish [16] and nPT in* Xenopus* [17]. The ADt neurones projected axons to pioneer the AC in which axons crossed the anterior midline, dorsal to the olfactory pits. The SOT axons also projected from a population of neurones located within the ADt and projected axons ventrally to join the TPOC (Figure 3(b)). The tract of the habenular commissure (THC) projected axons from the telencephalon to the dorsal diencephalon to form the habenular commissure ([16]; Figures 3(a) and 3(b). Due to the density of neurones and axons at this advanced stage, it was difficult to determine the origin of some of these later tracts. An unknown axon tract (1) was observed projecting along a curved route rostral towards the MLF (Figures 3(a)–3(c)). It was unclear where the neurones were located but they appeared to be located ventrally in the hypothalamus (Figure 3(b), arrowhead) and projected axons dorsally before turning along a more caudal route. In the caudal hypothalamus, a population of neurones appeared in a fan-like shape and projected axons caudally into the MLF and were possibly homologous to the mammillotegmental tract (MTT) (Figure 3(c), arrow). The tract of the posterior commissure (TPC) projected along the DMB forming the PC at the dorsal midline (Figure 3(a)). However, it was unclear where the TPC neurones were located. The location of the TPC along the DMB would suggest that the MLF neurones were both diencephalic and mesencephalic by stage 25. The DVDT axon was likely to still be present at stage 25; however labelling of the tract was not clear in the overview image (Figure 3(a)). The VC formed across the ventral midline at the DMB, with most axons present in the mesencephalon (Figure 3(a)).

## 4. Discussion

**Figure 4:**
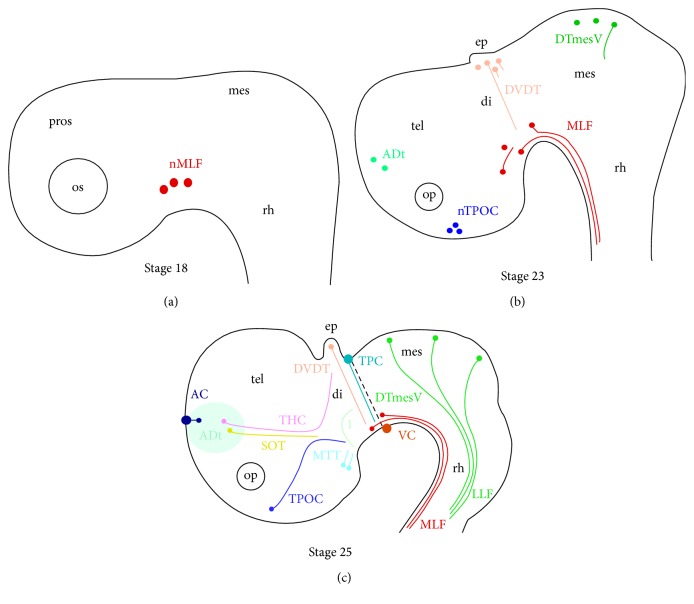
Developmental series of the* S. canicula* early axon scaffold. Schematic representation, with colour coding for the neurones and their associated axon tracts that arise in the* S. canicula* brain. (a) Stage 18: the appearance of the first neurones in the rostral brain. (b) Stage 23: MLF axons were projecting into the rhombencephalon, and the nTPOC, DTmesV, and DVDT have appeared. (c) Stage 25: the early axon scaffold appeared to be well established, with the addition of the AC, SOT, THC, TPC, VC, and LLF. AC: anterior commissure; ADt: anterior dorsal telencephalic neurones; di: diencephalon; DTmesV: descending tract of the mesencephalic nucleus of the trigeminal nerve; ep: epiphysis; LLF: lateral longitudinal fascicle; nMLF: nucleus of the medial longitudinal fascicle; nTPOC: nucleus of the tract of the postoptic commissure; mes: mesencephalon; MLF: medial longitudinal fascicle; MTT: mammillotegmental tract; op: olfactory pit; os: optic stalk; PC: posterior commissure; rh: rhombencephalon; SOT: supraoptic tract; tel: telencephalon; THC: tract of the habenular commissure; TPC: tract of the posterior commissure; TPOC: tract of the postoptic commissure; VC: ventral commissure.

We have presented a time series for the development of the initial axon tracts that form in the rostral* S. canicula* embryonic brain. Eight main tracts were observed: the MLF, TPOC, DVDT, DTmesV, TPC, MTT, SOT, and THC as well as three commissures: the PC, VC, and AC (Figure 4(c)). This basic setup of the first axon tracts from a small number of neurones in the embryonic brain was very similar to the early axon scaffold characterised in other vertebrates. Together with similar studies in the lamprey [2] and mouse [4, 6], these results confirm the ancient, conserved scaffold that appears in the vertebrate brain.

### 4.1. Initial Neurones That Differentiated in the* S. canicula* Brain

The MLF neurones were the first to appear in the embryonic brain at stage 18 (Figure 4(a)). The MLF remained the most prominent tract throughout much of the early axon scaffold development, until stage 23 when other neurones started to differentiate (Figure 4(b)). The appearance of the MLF neurones first is in line with many other vertebrates [1–5, 18].

In the absence of an accurate description of the molecular and anatomical subdivisions of the embryonic cat shark brain, the precise location of the neurones and their associated tracts was difficult to determine accurately. However, as the TPC projects along the DMB in mouse [4] and chick [5], it was likely that the transversal projection of the TPC axons in the diencephalon/mesencephalon region of the* S. canicula* brain also aligns with this boundary (Figure 4(c)). The location of the MLF neurones was more difficult to determine as in chick and zebrafish the neurones are strictly diencephalic [5, 19], whereas in mouse the neurones are both diencephalic and mesencephalic [4]. At later stages of* S. canicula* development, the MLF neurones have been shown to be strictly within prosomere 1 [11].

### 4.2. Comparison of Early Axon Scaffold Formation in* S. canicula* with Other Vertebrates

The early axon scaffold of* S. canicula* characterised here shares a number of key features with its counterpart in jawless (lamprey) and jawed vertebrates. The development of the early scaffold starts with the formation of the MLF, as in most vertebrates. Further tracts like the TPOC, TPC, SOT, and VC that could be identified in the embryonic cat shark brain are found in all vertebrates [2], indicating that they represent structures of the last common vertebrate ancestor.

Along with these similarities there were also differences. A noticeable feature of the* S. canicula* (and other cat shark species) brain was the distinct brain vesicles including an enlarged telencephalon, making its brain structure more similar to that of the amniotes rather than the anamniotes. A possible reason for this could be that when the cat shark hatches they are more developed as they have a longer developmental period compared with anamniotes such as zebrafish or* Xenopus* and do not have a larval stage.

One striking difference in axon tract development was the projection of the TPOC axons (Figure 4(c)). While the TPOC neurones were present in the hypothalamus like in other vertebrate species, the TPOC axons do not contribute to the ventral longitudinal tract (VLT) in these early stages. In chick, the VLT is composed of the TPOC, MTT, and MLF; this feature is also highly conserved [5]. While the TPOC tract formed from the nTPOC located in the rostral hypothalamus, the route the TPOC axons took in the shark was different to that analysed in other vertebrates [4, 5]. By stage 25, a tract homologous to the MTT was identified (Figure 4(c)). These neurones appeared to be in the caudal hypothalamus, where they were also located in chick and mouse [5]. The eventual shape of the neurone population was very different, forming a fan-like shape (Figure 4(c)). In the most rostral part of the brain, the POC formed from nTPOC neurones and these axons cross the midline in anamniotes [1]. The POC itself was not visible in the* S. canicula* stages analysed here but has previously been identified in the* S. torazame* brain [13]. Compared with anamniotes, the amniotes have fewer commissures during initial development although the amniotes eventually form these but at later stages. During early development,* S. canicula* forms the VC, PC, and AC (Figure 4(c)). While the VC and PC were also present in the initial early axon scaffold in chick and mouse [4, 5], the AC does not form until much later in chick [20] and mouse [21]. In anamniotes, the AC, SOT, and THC form from subpopulations within the large population of neurones located in the telencephalon termed the ADt (Figure 4(c)). No equivalent cluster of neurones is apparent in the early rostral brain of amniotes.

In anamniotes, the DTmesV forms much later in development [22, 23], while interestingly in* S. canicula* the DTmesV formed in the mesencephalon early (Figures 4(b) and 4(c)), reminiscent of amniotes [4, 5]. The DTmesV axons also took a slightly different route to that seen in the chick and mouse: the DTmesV axons did not project as far ventral into the mesencephalon alar plate in* S. canicula*, instead the axons projected along a caudal path to pioneer the LLF.

The SOT formed during initial development of the early axon scaffold in* S. canicula* and other anamniotes but later in other amniotes [24, 25]. The THC projects from the ventral diencephalon to form the habenular commissure (Figure 4(c)). In zebrafish, the THC appears around 28–30 hpf, after initial formation of the early axon scaffold, adding further evidence that at stage 25 the* S. canicula* early axon scaffold was well established [16]. The THC possibly contained stria medullaris (SM) axons as well, as these axons follow the same route [26].

In addition to the* S. torazame* description [13] we identified the VC, MTT, and an unidentified tract 1 (Figure 4(c)). This unidentified tract 1 could be homologous to A13 neurones present in the mouse [25], though tyrosine hydroxylase immunoreactivity has been reported to start at stage 26 [9, 11, 27], while unidentified tract 1 was well visible already by stage 25.

### 4.3. Conclusions

Our analysis highlights the conservation of the early axon scaffold as the first functional structure in the embryonic vertebrate brain. The overall organisation of the cat shark axon tracts is similar to that described for other vertebrates, including the early development of the medial longitudinal fascicle. Future work will involve lipophilic dye labelling of individual tracts to determine where neuronal populations are located and the mapping of the neuronal clusters against the molecular pattern of the embryonic brain, for example, the* Pax6* expression domain marking the DMB [28].
